# Genetic Variation in Spatio-Temporal Confined USA300 Community-Associated MRSA Isolates: A Shift from Clonal Dispersion to Genetic Evolution?

**DOI:** 10.1371/journal.pone.0016419

**Published:** 2011-02-04

**Authors:** Neeltje Carpaij, Rob J. L. Willems, Thomas W. Rice, Robert A. Weinstein, Jason Hinds, Adam A. Witney, Jodi A. Lindsay, Marc J. M. Bonten, Ad C. Fluit

**Affiliations:** 1 Department of Medical Microbiology, University Medical Centre Utrecht, Utrecht, The Netherlands; 2 Division of Infectious Disease, John H. Stroger Jr. Hospital of Cook County Chicago, Chicago, Illinois, United States of America; 3 Department of Infectious Diseases, Rush University Medical Center, Chicago, Illinois, United States of America; 4 Bacterial Microarray Group, Department of Cellular and Molecular Medicine, St George's University of London, London, United Kingdom; 5 Department of Cellular and Molecular Medicine, St. George's University of London, London, United Kingdom; National Institute of Allergy and Infectious Diseases, National Institutes of Health, United States of America

## Abstract

**Introduction:**

Community-associated methicillin-resistant *Staphylococcus aureus* (CA-MRSA) are increasingly isolated, with USA300-0114 being the predominant clone in the USA. Comparative whole genome sequencing of USA300 isolates collected in 2002, 2003 and 2005 showed a limited number of single nucleotide polymorphisms and regions of difference. This suggests that USA300 has undergone rapid clonal expansion without great genomic diversification. However, whole genome comparison of CA-MRSA has been limited to isolates belonging to USA300. The aim of this study was to compare the genetic repertoire of different CA-MRSA clones with that of HA-MRSA from the USA and Europe through comparative genomic hybridization (CGH) to identify genetic clues that may explain the successful and rapid emergence of CA-MRSA.

**Materials and Methods:**

Hierarchical clustering based on CGH of 48 MRSA isolates from the community and nosocomial infections from Europe and the USA revealed dispersed clustering of the 19 CA-MRSA isolates. This means that these 19 CA-MRSA isolates do not share a unique genetic make-up. Only the PVL genes were commonly present in all CA-MRSA isolates. However, 10 genes were variably present among 14 USA300 isolates. Most of these genes were present on mobile elements.

**Conclusion:**

The genetic variation present among the 14 USA300 isolates is remarkable considering the fact that the isolates were recovered within one month and originated from a confined geographic area, suggesting continuous evolution of this clone.

## Introduction

The epidemiology of methicillin-resistant *Staphylococcus aureus* (MRSA) infections has changed dramatically during the last 15 years. While traditionally MRSA was a typical example of a nosocomial pathogen, it is now frequent found as causative agent of community-associated infections among patients without known risk factors for hospital-acquired (HA)-MRSA [1], [2], [3], [4], [5], [6], [7].

The molecular epidemiology of community-associated MRSA (CA-MRSA) is diverse, although certain clones appear to dominate on every continent. These predominant CA-MRSA clones cluster in different lineages: ST8/USA300, ST1/USA400, ST30/USA1100, ST93, ST59, ST80 and ST398 clone [8].

In the USA, the first widely recognized CA-MRSA clone was USA400 (ST1). After the turn of the century, USA300 (ST8) emerged rapidly across the USA and replaced USA400 as the dominant clone in the USA responsible for the majority of skin and soft tissue infections. Subsequently, USA300 has been increasingly isolated outside the USA indicating pandemic spread. Within USA300 several subtypes exist of which PFGE-type USA300-0114 predominates [9], [10].

Comparative whole genome sequencing of 10 USA300 CA-MRSA and HA-MRSA isolates collected nationwide in the USA in 2002, 2003, and 2005 showed a limited number of single nucleotide polymorphisms and regions of differences among USA300 isolates, which suggests that USA300 has undergone rapid clonal expansion without great genomic diversification [11]. So far whole genome comparisons of CA-MRSA are limited to isolates belonging to USA300. The aim of this study was to compare the genetic repertoire of different CA-MRSA clones with that of HA-MRSA from the USA and Europe through comparative genomic hybridization (CGH) to identify genetic clues that may explain the successful and rapid emergence of CA-MRSA [12].

## Materials and Methods

### Bacterial isolates and nucleic acid extraction

Thirty nine consecutive MRSA isolates collected in 2004, originating from hospitalized patients with serious invasive infections admitted at Cook County Hospital (Chicago IL, USA) were taken from a database. The isolates were phenotypically classified with MicroScan (West Sacramento, California) as methicillin-resistant. Twenty isolates were recovered <48 h after admittance from patients who did not have prior health care exposure and were considered CA-MRSA. These isolates were collected between the 1^st^ and 28^th^ of July. Nineteen isolates were recovered (between July and October of 2004) from patients >48 h after admission and these were classified as HA-MRSA (Table S1).

Ten HA-MRSA isolates from The Netherlands, Germany, Belgium, and France were selected from a collection of 118 HA-MRSA isolates present at the University Medical Center Utrecht (UMCU) on the basis of their geographic origin, infections caused or nasal carriage and multilocus sequence type (ST) (Table S1). In addition, one CA-MRSA isolate, obtained in 2001 from a child that succumbed due to necrotizing pneumonia within 48 h of hospitalization (and without prior health care exposure) in the UMCU [13]. All isolates were cultured overnight on tryptic soy agar with sheep blood at 37°C. DNA was extracted with a Nucleospin Tissue kit according to manufacturer's protocol (Bioké, Leiden, The Netherlands). Plasmid DNA content was determined using S1 nuclease treatment (Takara Bio Europe, Saint-Germain-en-Laye, France), gel electrophoreses and southern blot with a 193 bp probe from the resolvase gene SAR719.

We do not have an ethics approval and an informed consent, because the data we have used are anonymous and were not specifically collected for our study. The patients were admitted to the hospital, the samples were taken for diagnostic purposes by the treating physicians in order to appropriately treat the patients. These samples were anonymously put in a database from which they were recovered. In the Netherlands the Medical Ethical Committee does not require an approval for this kind of research and sampling.

### Typing of the MRSA isolates

Colony morphology and standard techniques, like multiplex PCR for the 16S rRNA gene, *mecA* gene, and *nuc* gene were used to confirm whether the isolates were MRSA. All MRSA isolates from the USA were typed by MLST [14]. The CA-MRSA isolates were further characterized by PFGE, *spa*, SCC*mec* and antimicrobial susceptibility patterns by MicroScan (Dade Behring Inc., West Sacramento, CA, USA) [15], [16], [17]. The antimicrobial agents tested were oxacillin, erythromycin, rifampicin, fluoroquinolones, clindamycin, gentamicin and tetracycline. The MIC values were interpreted according to the Clinical and Laboratory Standards Institute recommendations (2004) [18].

### Comparative genomic hybridization

Gene content of the CA- and HA-MRSA isolates was determined via CGH using a previously described multistrain (n = 7) PCR product *S. aureus* microarray [19], [12]. The array design is available in BµG@Sbase (Accession No. A-BUGS-17; http://bugs.sgul.ac.uk/A-BUGS-17) and also ArrayExpress (Accession No. A-BUGS-17). Gene presence and divergence was determined using the algorithm described previously [20]. Complete linkage hierarchical clustering with Euclidian distance of all the HA-MRSA and CA-MRSA isolates was used to visualize the genetic relatedness.

### Confirmation of genetic differences

Differences in gene content between the USA300 CA-MRSA isolates from Chicago and USA300FPR3757 and USA300TCH1516 (for which the whole genome sequences are available) as identified by CGH were confirmed by PCR and sequencing. The primers were generated, if possible, using the USA300FPR3757 sequence and otherwise on the USA300TCH1516 or MRSA252 sequence. Amplification was carried out for 35 cycles with denaturation at 95°C for 30 seconds, annealing at the primer specific annealing temperature for 30 seconds, extension at 72°C for 45 seconds and a final extension at 72°C for 7 minutes. The presence of *arcA* considered specific for SCC_ACME_, *lukS-PV* and *lukF-PV,* encoding Panton-Valentine leukocidin, *sek* and *seq* encoding enterotoxin K and Q, respectively, in the USA300 strains from Chicago were determined by PCR as previously described [9], [21]. The primers and conditions used for confirmation are shown in the Table S2.

## Results

### Molecular epidemiology of CA- and HA-MRSA

The majority of the 19 HA-MRSA isolates from the USA belonged to ST5 (n = 14, 74%). From the five other isolates, four were classified as ST8 and one as ST45 (Table S1).

**Figure 1:**
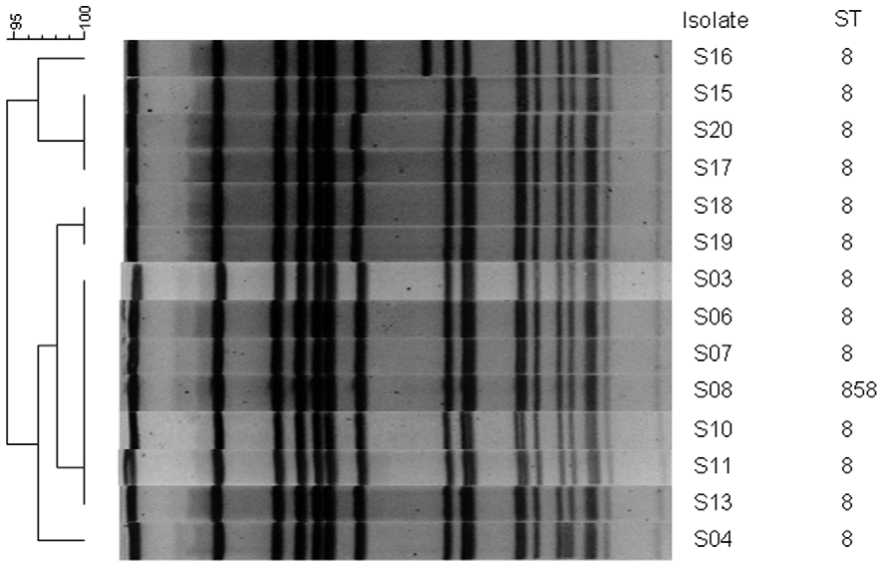
PFGE pattern of the 14 USA300 strains. PFGE pattern clusters of 14 USA300 isolates from Chicago clustered using BioNumerics version 5.10 (Applied Maths NV, Sint-Martens-Latem, Belgium). Isolate number and ST are indicated. The left side of the banding pattern indicates the start of the PFGE gel. Thirteen of the 14 isolates belong to PFGE type USA300-0114, only S16 showed two bands difference and belongs to a different subtype.

Two of the 20 CA-MRSA isolates from the USA were *mecA* negative in the PCR, and were no longer considered MRSA. From the remaining 18 CA-MRSA isolates from the USA, 14 were typed by PFGE as USA300 and 13 represented the USA300-0114 subtype. One isolate (S16) had a slightly different PFGE subtype and differed by two bands (Figure 1). MLST was in agreement with these data, since 13 USA300 strains were ST8. One isolate (S08) represented ST858, a single locus variant of ST8 (Table 1). All isolates, except one, belonged to *spa*-type t008. That isolate (S06) had a new *spa*-type, t4913 (11-19-36-21-17-34-24-34-22-25), one repeat difference with t008 (Table 1).

**Table 1:** Demographic and clinical characteristics of the 18 CA-MRSA isolates from the USA based on MicroScan.

Isolate	Isolation date	PFGE type	ST (CC)	*spa*-type	SCC*mec*	Isolation site	Resistance^a^
S01	07-02-2004	USA100 (subtype b)	5	t002	II	Blood	β,CD, L
S02	07-02-2004	USA100 (subtype a)	5	t002	II	Skin/soft tissue	β,CD, L
S03	07-04-2004	USA300-0114	8	t008	IV	Skin/soft tissue	β,E
S04	07-04-2004	USA300-0114	8	t008	IV	Blood	β
S05	07-03-2004	USA400 (subtype c)	1	t008	IV	Skin/soft tissue	β
S06	07-07-2004	USA300-0114	8	t4913	IV	Skin/soft tissue	β,E
S07	07-06-20 04	USA300-0114	8	t008	IV	Skin/soft tissue	β,E
S08	07-09-2004	USA300-0114	858 (8)	t008	IV	Skin/soft tissue	β,E
S10	07-12-2004	USA300-0114	8	t008	IV	Skin/soft tissue	β,E
S11	07-16-2004	USA300-0114	8	t008	IV	Skin/soft tissue	β,E
S12	07-17-2004	USA100 (subtype b)	5	t002	II	Cathether	β,E,L
S13	07-16-2004	USA300-0114	8	t008	IV	Skin/soft tissue	β
S15	07-20-2004	USA300-0114	8	t008	IV	Skin/soft tissue	β,E
S16	07-22-2004	USA300	8	t008	IV	Blood	β,E,R
S17	07-24-2004	USA300-0114	8	t008	IV	Skin/soft tissue	β,E,T
S18	07-26-2004	USA300-0114	8	t008	IV	Skin/soft tissue	β,E
S19	07-28-2004	USA300-0114	8	t008	IV	Skin/soft tissue	β,E
S20	07-28-2004	USA300-0114	8	t008	IV	Skin/soft tissue	β,E,T

a Resistance patterns were determined with MicroScan (Dade Behring INC. West Sacramento, CA, USA). β = β-lactam antbiotics, E =  erythromycin, R =  rifampicin, L =  quinolones, CD =  clindamycin, G =  gentamicin and T =  tetracycline.

The other four CA-MRSA from the USA belonged to PFGE type USA400 (ST1) (n = 1) and USA100 (ST5) (n = 3) (Table 1). The CA-MRSA isolate from The Netherlands belonged to ST80.

The infections caused by the HA-MRSA isolates were more diverse than the infections caused by the CA-MRSA strains (Table S1). The patients with HA-MRSA suffered from pneumonia (n = 7), skin and soft tissue infections (n = 7), urinary tract infections (n = 3), sepsis (n = 8) and a bone infection (n = 1). Three isolates came from colonized patients and were collected with nasal swabs (Table S1). The majority of the CA-MRSA isolates from the USA (n = 14, 78%) caused skin and soft tissue infections. In three patients (S01, S04 and S16), the bacteria were isolated from blood. In one other (S12) case the bacteria were isolated from a catheter (Table 1).

Two of the 14 USA300 CA-MRSA isolates (S04 and S13) were only resistant to β-lactam antibiotics (Table 1). The other 12 were also resistant to erythromycin. One isolate (S16) was also resistant to rifampicin.

### Differences in gene content between CA- and HA-MRSA

**Figure 2:**
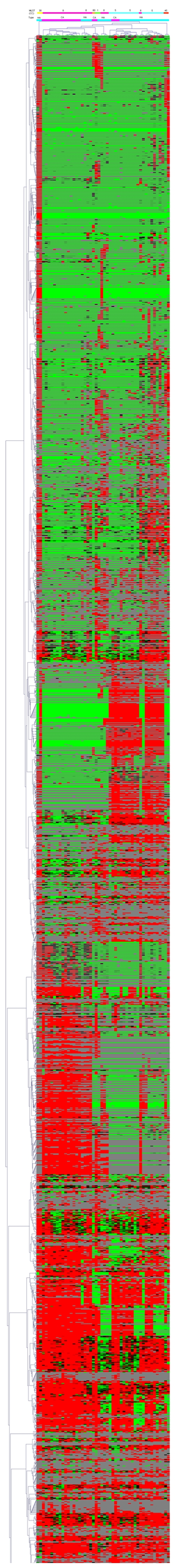
Hierarchical clustering of HA and CA-MRSA from the USA and the Netherlands. Hierarchical clustering based on Euclidean distance with complete linkage made in TIGR MultiExperiment Viewer version 3.1 (Dana-Farber Cancer Institute, Boston, USA). The genes are clustered on the y-axis. On the x-axis the fifty different CA-MRSA and HA-MRSA isolates from the USA and the Netherlands are depicted. In this figure the conserved genes are not depicted, although they haven been used to for clustering. The top colored line depicts the different ST-types: pink bars: CC8, light blue bars: CC5, yellow bars: ST30, orange bar: ST80, green bars: ST1, red bars: ST45. The second colored bar represents the different MRSA-types: pink bars: CA-MRSA; light blue bars indicate HA-MRSA.

Fully annotated microarray data have been deposited in BµG@Sbase (accession number E-BUGS-108; http://bugs.sgul.ac.uk/E-BUGS-108). Hierarchical clustering based on the CGH-data showed no grouping of isolates based on community or hospital origin but on the basis of their ST type, which is in concordance with previous observations. Isolates with ST5 and -8 are split in different groups (Figure 2) [19]. The 19 CA-MRSA isolates were dispersed among all isolates represented on the dendogram (Figure 2). This finding indicates that the CA-MRSA do not share a common and unique genetic repertoire. The PVL genes were present in all CA-MRSA isolates and absent in all HA-MRSA isolates based on the microarray data and confirmed by PCR. In addition, PCR demonstrated that *arcA, sek* and *seq* were present in all analyzed USA300 CA-MRSA.

### Genomic variation among USA300 CA-MRSA

There were remarkable genetic differences between the 14 USA300 CA-MRSA isolates. A difference in gene content was not confined to one isolate but was found in nine of the 14 isolates (Table 2), although isolate S04 had most variation compared to the other isolates. Furthermore, differences in plasmids were found among the 14 isolates from the USA. Although most isolates (n = 9, 65%) contained two plasmids of similar size (data not shown), four isolates did not contain any plasmids (S3, S6, S15 and S18) and one isolate (S20) had three plasmids, which were of the same size as the plasmids of USA300FPR3757.

**Table 2:** Genetic differences between the 14 USA300 isolates based on comparative genomic hybridization data and PCR.

Gene	MRSA252 (ORF)	Location	Results for															
			S3	S4	S6	S7	S8	S10	S11	S13	S15	S16	S17	S18	S19	S20	USA300 TCH1516	USA300 FPR3757
DNA repair protein	SAR0617	MGE	+	+	+	+	+	+	+	+	+	+	-	+	+	+	+	+
Resolvase	SAR0719	MGE	+	-	+	+	+	+	+	+	+	+	+	+	+	+	-	+
Serine protease-like C	SAR1906	Chromosome	-	+	-	+	+	+	-	+	+	+	+	+	+	+	+	+
Serine protease-like E	SAR1902	Chromosome	-	+	+	+	+	+	+	+	+	+	+	+	+	+	+	+
Membrane protein	SAR2132	MGE	+	+	+	+	+	+	+	+	+	+	+	-	+	+	+	+
Hypothetical protein	SAR1682	MGE	+	-	+	+	+	+	+	+	+	+	+	+	+	+	-	-
Phage protein	SAR1554	MGE	-	+	-	-	-	-	-	-	-	-	-	-	-	-	+	+
Phage protein	SAR2066	MGE	+	+	+	+	+	+	+	-	+	+	+	+	+	+	+	+
Exported protein (PCR)^a^	SAR2565	Chromosome	+	-	+	+	+	+	+	+	-	+	+	+	+	+	-	-
Exported protein (microarray)^b^	SAR2565	Chromosome	NR^c^	-	NR	NR	NR	+	NR	NR	NR	NR	NR	-	+	-	-	-
Hypothetical protein	SAR0056	MGE	+	+	+	+	+	+	+	+	+	+	+	+	+	-	+	+

a The results of the Exported gene (SAR2565) in the confirmation PCR, of which the primers are shown in Table S2.

b The results of the Exported gene (SAR2565) gained by the hybridization of the 14 USA300 isolates on the microarray earlier described.

c NR: not reliable.

CGH (with PCR confirmation) indicated that nine genes were variably present among the 14 USA300 strains (Table 2). In addition, one gene present in the sequenced USA300FPR3575 and USA300TCH1516 strains was absent in all 14 analyzed USA300 CA-MRSA isolates. This gene (SAR2565) encodes an exported protein (Table 2).

Seven of the in total 10 genes that were variably present or absent in USA300 isolates, were located on known mobile genetic elements (MGE) in the published genomes (Table 2), whereas the three other genes were located in regions that are not considered to be mobile. This indicates that the genes that are variably present in USA300 are not located adjacent to each other, but dispersed across the genome (data not shown).

The 10 genes encode for serine protease-like E protein (*splE*) (SAR1902), serine protease-like C protein (*splC*) (SAR1906); a resolvase (*tnpR*) (SAR0719), a putative DNA repair protein (SAR0617), the exported protein gene SAR2565, a gene encoding a membrane protein (SAR2132), two genes encoding phage proteins (SAR1554 and SAR2066) and two genes encoding hypothetical proteins (SAR1682 and SAR0056).

The differentially present genes *splE* and *splC* are part of a 5 kb *spl* operon, encoded on genomic island νSaβ which is present in most, but not in all *S. aureus* strains [22]. The other four genes of the *spl-*operon (*splA, splB, splD,* and *splF*) were present in all USA300 isolates analyzed by CGH.

The resolvase gene (SAR719), which is absent in isolate S04, is located on a 26 kb plasmid (pUSA300HOUMR) in USA300TCH1516. This was confirmed by Southern blot (data not shown).

The membrane protein gene (SAR2132), absent in isolate S18 has 58.62% (amino acid) similarity with the hypothetical protein YeeE in *E. coli* which is a putative transport system permease protein. This gene is present in the majority of the sequenced *S. aureus* strains (data not shown).

There were two genes that were differentially present and that tentatively encode phage proteins. One of the hypothetical phage proteins (SAR2066) is similar to the C-terminal region of *Streptococcus thermophilus* bacteriophage Sfi21 hypothetical protein and is only present in the sequenced USA300 isolates, MRSA252, Mu3 and Newman strains. The second phage protein (SAR1554) is 97.96% (amino acid) similar to a *S. aureus* prophage phiPV83 protein which is differently present in the sequenced strains MRSA252, JH9, JH1 and S0385**.**

## Discussion

The rapid emergence of specific MRSA clones, like USA300, first in the community followed by dispersal in hospitals is still largely unexplained. Our study confirmed previous findings that all CA-MRSA clones do not share a common genetic repertoire that is unique to these clones other than PVL toxin genes, which are present in most USA300 isolates [23], [21], [24]. Despite being ubiquitously present in our set of USA300 isolates, USA300 isolates without the PVL and SCC_ACME_ genes have been described recently [25], [26]. This could mean that PVL and probably SCC_ACME_ have a less significant role in CA-MRSA virulence than previously assumed.

In contrast to previously published studies by Tenover et al. and Kennedy et al., we found an unexpected high number of genetic differences among 13 PFGE subtype USA300-0114 clones isolated within the time-frame of one month from a single location [11], [27]. Of the genes that were differentially present among the USA300 isolates from Chicago, or even completely absent, compared to the sequenced USA300 isolates, eight were identified for the first time as being differentially present among CA-MRSA, thus representing non-core CA-MRSA genes. However, it should be noted that divergent gene orthologues that are not represented on the array, may be present among the 14 USA300 isolates because USA300 was not used to create the microarray. Next to that the majority of the sequenced isolates used to generate the microarray described in this study were laboratory strains, which were collected years before the clinical USA300 specimens were accumulated, making it even more likely that USA300 strains and other CA-MRSA clones may contain novel genetic elements absent in the older strains.

Non-core genes contribute significantly to the overall diversity of gene repertoires in a species. The vertically transmitted core genome encodes fundamental cellular processes, and the horizontally transmissible accessory genome encodes for a variety of secondary metabolites, resistance to specific toxins, virulence factors or antibiotics [28]. Seven of the in total 10 genes that were variably present or absent in USA300 isolates, were located on mobile genetic elements (MGE) (Table 2). This was as expected, because mobile genetic elements easily account for significant gene diversity among strains that are closely related with respect to the core genome. However, 3 of the 10 genes were located in regions that are not considered to be MGEs. In addition, the genetic differences, which are located on the MGEs involve only single genes and the isolates did not lose or acquire a complete genetic element. But we also found variation in the plasmid content of the 14 USA300 isolates.

From literature (Kennedy et al. and Tenover et al.) it seems that the USA300 isolates used in these studies [11], [27] were less diverse. The majority of these isolates are collected from different geographic regions and before 2004. This could suggest the USA300 isolates are less genetically diverse before 2004. However, in order to be certain about this, it will be necessary to analyze isolates from the same hospital in Chicago before 2004. Unfortunately we do not have these data.

Two of the variably present genes are located on the *spl*-operon. The *spl* genes are positively controlled by *agr,* one of the regulators responsible for the global regulation of virulence factors in *S. aureus,* which is conserved in all staphylococcal species. The genes are most similar to a V8 protease, which can cleave the heavy chain of human immunoglobulin classes in vitro. In 64% of the isolates the s*pl* operon was present and no obvious role in virulence was demonstrated in intraperitoneally injected rats [22], [29].

The observed genetic differences between the 14 USA300 isolates, recovered within the time-frame of one month and from a single location, suggests continuous evolution of this clone. In fact one may argue that following a period of intense and rapid clonal dispersion, USA300 has now entered a period of development of a polyclonal lineage rather than a single dispersing clone, which was also suggested by Kennedy et al [11]. Whether the observed differences in gene content indicate random loss or acquisition of genes or reflect local adaptation, and whether these differences affect virulence remains to be investigated.

## Supporting Information

Table S1 **Characteristics of HA-MRSA isolates used in this study.** ^a^ Sequence type (ST) with clonal complex between brackets.
(DOC)

Table S2 **PCR primers used to validate the 10 genetic differences.** ^a^ MRSA252 ORF: MRSA252 open reading frame is the MRSA252 gene-number, which corresponds with the specific gene.
(DOC)
