# Supplementary material for: Genetic Variation in Spatio-Temporal Confined USA300 Community-Associated MRSA Isolates: A Shift from Clonal Dispersion to Genetic Evolution?
Source: PLoS One. 2011 Feb 4;6(2):e16419. doi: 10.1371/journal.pone.0016419 (PMC3033884; doi:10.1371/journal.pone.0016419)
Supplement: Table S1 — Characteristics of HA-MRSA isolates used in this study. a Sequence type (ST) with clonal complex between brackets. (DOC) [file pone.0016419.s001.doc]

**Supplementary Table 1. Characteristics of HA-MRSA isolates used in this study.**

| Isolate | ST (CC) | Isolation site | Origin |
| --- | --- | --- | --- |
| S23 | 45 | Blood | Belgium, Brussels |
| S29 | 8 | Blood | France, Paris |
| S35 | 247 (CC8) | Blood | France, Lille |
| S57 | 225 (CC5) | Blood | Germany, Munich |
| S110 | 247 (CC8) | Hip-bone | the Netherlands, Utrecht |
| S112 | 5 | Nose (carriage) | the Netherlands, Utrecht |
| S113 | 36 (CC30) | Nose (carriage) | the Netherlands, Utrecht |
| S119 | 188 (CC1) | Skin/soft tissue | the Netherlands, Utrecht |
| S159 | 30 | Nose (carriage) | the Netherlands, Utrecht |
| S162 | 239 (CC8) | Blood | Greece, Athens |
| S948 | 5 | Respiratory tract | USA, Chicago |
| S949 | 8 | Respiratory tract | USA, Chicago |
| S950 | 5 | Respiratory tract | USA, Chicago |
| S951 | 5 | Respiratory tract | USA, Chicago |
| S952 | 5 | Catheter | USA, Chicago |
| S953 | 5 | Catheter | USA, Chicago |
| S954 | 5 | Respiratory tract | USA, Chicago |
| S955 | 5 | Skin/soft tissue | USA, Chicago |
| S956 | 8 | Skin/soft tissue | USA, Chicago |
| S957 | 5 | Urinary tract | USA, Chicago |
| S958 | 5 | Skin/soft tissue | USA, Chicago |
| S959 | 8 | Catheter | USA, Chicago |
| S960 | 5 | Urinary tract | USA, Chicago |
| S961 | 5 | Skin/soft tissue | USA, Chicago |
| S962 | 5 | Respiratory tract | USA, Chicago |
| S963 | 45 | Skin/soft tissue | USA, Chicago |
| S964 | 5 | Skin/soft tissue | USA, Chicago |
| S965 | 5 | Urinary tract | USA, Chicago |
| S967 | 8 | Respiratory tract | USA, Chicago |
