# Supplementary material for: Genetic Variation in Spatio-Temporal Confined USA300 Community-Associated MRSA Isolates: A Shift from Clonal Dispersion to Genetic Evolution?
Source: PLoS One. 2011 Feb 4;6(2):e16419. doi: 10.1371/journal.pone.0016419 (PMC3033884; doi:10.1371/journal.pone.0016419)
Supplement: Table S2 — PCR primers used to validate the 10 genetic differences. a MRSA252 ORF: MRSA252 open reading frame is the MRSA252 gene-number, which corresponds with the specific gene. (DOC) [file pone.0016419.s002.doc]

**Supplementary Table 2. PCR primers used to validate the 10 genetic differences.**

| Gene encoding for | MRSA252 ORF | Validation PCR |  |  |
| --- | --- | --- | --- | --- |
|  |  | Primer Forward | Primer Reverse | Product Length (bp) |
| DNA repair protein | SAR0617 | aattttggtggcctgctgataa | tgaaatgatgtcgtgctaacc | 245 |
| Resolvase | SAR0719 | tgaatttgcaaggtctggag | aaatgctgcgaataaatgaaat | 193 |
| Serine protease like C | SAR1906 | ttcccgggttcaatgta | atataatggcgtcgttca | 426 |
| Serine protease like E | SAR1902 | caatttccatacctgcaacgacat | aacaaacggctaaagctgaacata | 210 |
| Membrane protein | SAR2132 | acggaatgccagttgtttga | tcgggggactcttaggtttt | 426 |
| Hypothetical protein | SAR1682 | cgttaatttttcagtcgcatcc | cgcctcagccgttcaaaatc | 322 |
| Phage protein | SAR1554 | gctgctaggttgtattcact | tcatccgattccgctaa | 158 |
| Phage protein | SAR2066 | cggatgatgttctagcgataat | ctaagaatggcaaacaacaag | 148 |
| Exported protein | SAR2565 | aattgatgcgctggtttta | cgcgcattacactttatct | 235 |
| Hypothetical protein | SAR0056 | gtcgcttcgcataggcaccat | gcaccgctatttactcaagaa | 305 |
